# Efficacy and safety of robotic surgery versus open surgery for hilar cholangiocarcinoma: a systematic review and meta-analysis

**DOI:** 10.1097/JS9.0000000000001952

**Published:** 2024-07-22

**Authors:** Manqin Hu, Dingwei Xu, Yan Zhang, Ao Li, Xincheng Li, Jie Huang

**Affiliations:** The Third Division of Department of Hepatobiliary and Pancreatic Surgery, The Second Affiliated Hospital of Kunming Medical University, Kunming, People’s Republic of China

**Keywords:** hilar cholangiocarcinoma, meta-analysis, open surgery, robotic surgery

## Abstract

**Objective:**

The aim is to assess and contrast the effectiveness and safety of employing robotic surgery versus traditional open surgery in managing cases of hilar cholangiocarcinoma.

**Methods:**

Computer searches were conducted in PubMed, Embase, Cochrane Library, Web of Science, China National Knowledge Infrastructure (CNKI), and Wanfang Database to identify case–control studies comparing robotic surgery with traditional open surgery in the treatment of hilar cholangiocarcinoma from inception until July 2023. References from retrieved articles were reviewed to broaden the search. This review was prospectively registered in the PROSPERO database (PROSPERO ID: CRD42024527511) and reported in line with PRISMA (Preferred Reporting Items for Systematic Reviews and Meta-Analyses) and AMSTAR (Assessing the methodological quality of systematic reviews) Guidelines. The primary outcome measures included operation time, intraoperative blood transfusion rate, R0 resection rate, lymph node metastasis rate, incidence of postoperative complications, and postoperative hospital stay. Data analysis was performed using RevMan 5.4 software, calculating combined odds ratios (OR), mean differences (MD), and 95% CI.

**Results:**

A total of 4 studies encompassing 267 patients diagnosed with hilar cholangiocarcinoma (177 males and 90 females, mean age of (58.8±5.7) years) were included in this analysis. Among these, 165 patients underwent open surgery, while 102 patients underwent robotic surgery. The results of the meta-analysis demonstrated comparable outcomes between the two groups. Specifically, the operation time between the robotic surgery and open surgery cohorts did not significantly differ (MD=−103.96, 95% CI: −216.90 to 8.98, *P*=*0.070*). Additionally, the intraoperative blood transfusion rate (OR=1.32, 95% CI: 0.43–4.07, *P*=0.630), R0 resection rate (OR=1.41, 95% CI: 0.71–2.81, *P=0.330*), and lymph node metastasis rate (OR=1.62, 95% CI: 0.46–5.63, *P=0.450*) showed no significant differences between the groups. Similarly, there were no statistically significant disparities observed in the incidence of postoperative complications (OR=0.60, 95% CI: 0.28–1.31, *P=0.200*) and postoperative hospital stay (MD=2.17, 95% CI: −11.56 to 15.90, *P=0.760*).

**Conclusion:**

In the treatment of hilar cholangiocarcinoma, robotic surgery demonstrates comparable safety and feasibility to traditional open surgery. However, due to the limited quantity and quality of the included studies, these conclusions warrant validation through additional high-quality investigations.

## Introduction

Highlights

- This meta-analysis is still a blank field at home and abroad, and our study has also been certified by the international meta-analysis inplasy platform.
- Different from previous laparoscopic and traditional surgery, our study focuses on the comparison between robot and traditional surgery, and has a deeper understanding of the efficacy and safety of minimally invasive surgery in the treatment of hilar cholangiocarcinoma.

Hilar cholangiocarcinoma (HCCA) is the most common subtype of cholangiocarcinoma (60–67%), characterized by high invasiveness and a poor prognosis^1,2^. Research indicates a 5-year survival rate of only 31.8% for patients with HCCA^3^. Hepatectomy, combined with complete extrahepatic bile duct resection, radical lymph node dissection, and biliary reconstruction, is the established standard procedure for managing HCCA^4^.

In 2011, Giulianotti *et al*.^5^ reported the increasing utilization of robotic surgery for hilar cholangiocarcinoma, highlighting its progressive adoption as a minimally invasive approach in liver tumor treatment. Studies^6^ have indicated that robot-assisted radical resection in hilar cholangiocarcinoma surpasses the limitations associated with traditional open surgery, notably mitigating substantial trauma and multiple complications. Currently, limited research exists on the comparative outcomes of robotic hand surgery and traditional open surgery in treating hilar cholangiocarcinoma. The meta-analysis systematically assesses the existing literature, examining and comparing the efficacy and safety of robotic surgery versus open surgery in managing hilar cholangiocarcinoma.

## Methods

### Protocol and registration

This review was prospectively registered in the PROSPERO database (PROSPERO ID: CRD42024527511, Supplemental Digital Content 1, https://kdocs.cn/l/cry8L7ePVkBq?f=201).This systematic review was reported following the Preferred Reporting Items for Systematic Reviews and Meta-Analyses (PRISMA) guidelines^7^(Supplemental Digital Content 2, https://kdocs.cn/l/cvIfj2xkiJrd?f=201, Supplemental Digital Content 3, https://kdocs.cn/l/ci6aDVbsxYHi?f=201) and Assessing the Methodological Quality of Systematic Reviews (AMSTAR) standards^8^ (Supplemental Digital Content 4, https://kdocs.cn/l/catHXScSdzof?f=201).

### Search strategy

**Figure 1:**
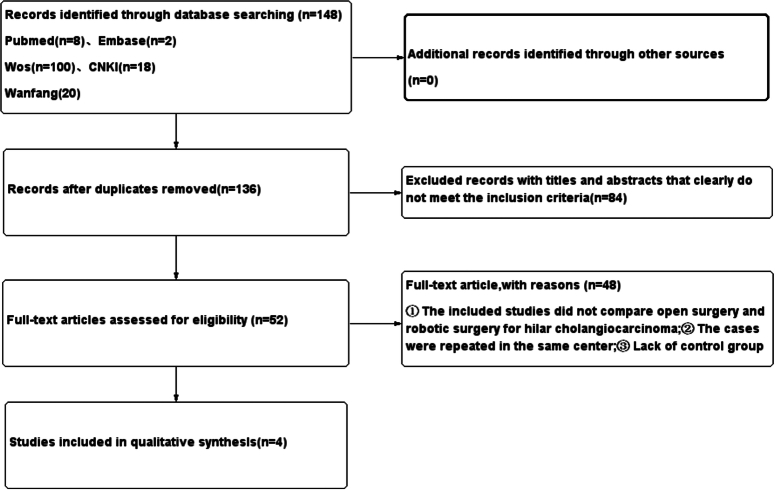
Retrieval process and results.

A comprehensive computerized search was conducted across multiple databases, including PubMed, Embase, Cochrane, Web of Science, CNKI, and Wanfang, spanning from the library’s inception to July 2023. This search encompassed studies in all languages, and we meticulously examined all eligible studies and their bibliographies to identify relevant research. The search terms employed were: (‘Robotic Surgical Procedures[Mesh]’ OR ‘Procedure, Robotic Surgical’ OR ‘Procedures, Robotic Surgical’ OR ‘Robot Surgery’ OR ‘Robotic Surgical Procedure’ OR ‘Surgical Procedure, Robotic’ OR ‘Robot Surgeries’ OR ‘Surgery, Robot’ OR ‘Robot-Assisted Surgery’ OR ‘Robot-Assisted Surgery’ OR ‘Robot-Assisted Surgeries’ OR ‘Surgery, Robot-Assisted’ OR ‘Robot-Enhanced Procedures’ OR ‘Procedure, Robot-Enhanced’ OR ‘Robot Enhanced Procedures’ OR ‘Robot-Enhanced Procedure’ OR ‘Surgical Procedures, Robotic’ OR ‘Robotic-Assisted Surgery’ OR ‘Robotic-Assisted Surgery’ OR ‘Robotic-Assisted Surgeries’ OR ‘Surgery, Robotic-Assisted’ OR ‘Robot-Enhanced Surgery’ OR ‘Robot Enhanced Surgery’ OR ‘Robot-Enhanced Surgeries’ OR ‘Surgery, Robot-Enhanced’) AND (‘open surgery[Mesh]’ OR ‘open operation’ OR ‘laparotomy’) AND (‘Klatskin tumor[Mesh]’ OR ‘cholangiocarcinoma Hilar’ OR ‘Hilar cholangiocarcinoma’) (Fig. 1).

### Study selection criteria

Inclusion criteria: (1) case–control studies comparing the efficacy of open surgery and robotic surgery in treating hilar cholangiocarcinoma; (2) patients with hilar cholangiocarcinoma (unlimited age, sex, nationality, etc.); (3) the outcome index includes at least one of the following: operation time, intraoperative blood transfusion rate, number of lymph node dissection, R0 resection rate, postoperative complications, hospital stay, and so on.

Exclusion criteria: (1) case reports, reviews, or conference reports. (2) Selection of the most recent publication when multiple publications report the same central case. (3) Studies of poor quality. (4) Studies lacking a control group. (5) Insufficient availability of experimental data.

### Literature screening, data extraction, and quality assessment

**Figure 2:**
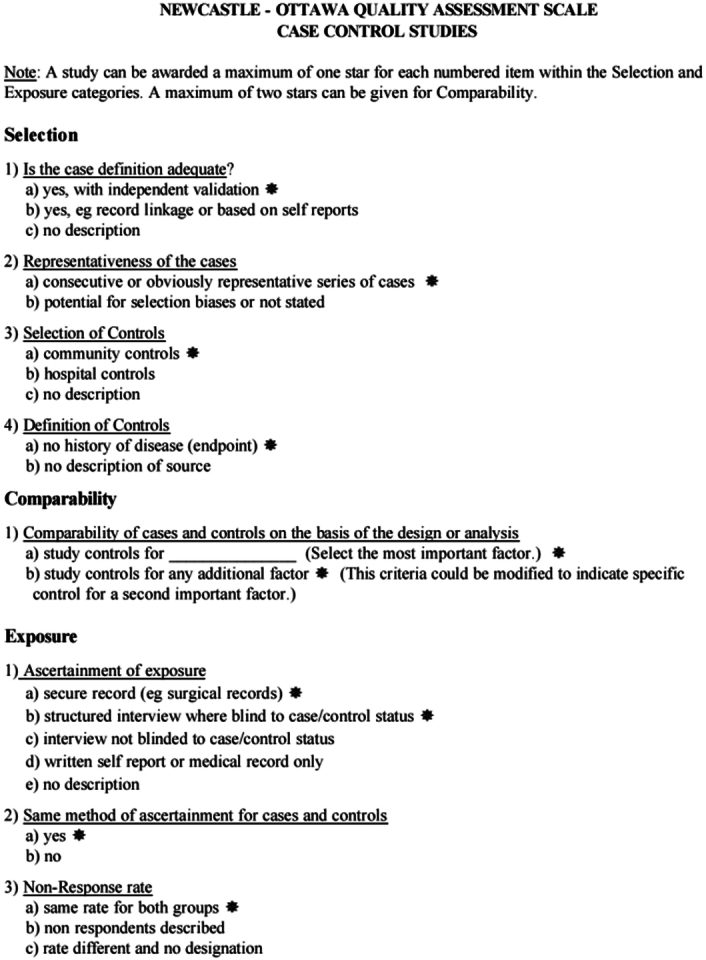
Newcastle-Ottawa-Scale for case-control studies.

Two researchers independently conducted literature screening, extracting data, and completing the quality evaluation. Initially, they reviewed the literature titles and abstracts, excluding irrelevant works, and further selected potentially relevant literature based on the inclusion-exclusion criteria. Extracted data included details such as first author, country, publication year, patient numbers, demographics, Bismuth–Corlette classification, operation time, lymph node metastasis rate, intraoperative blood transfusion rate, R0 resection rate, postoperative hospital stay, incidence of postoperative complications, and others. The quality evaluation was performed using the Newcastle–Ottawa Scale (NOS), (Fig. 2 and Table 1) where scores higher than 6 are deemed as high-quality^9^. Findings were cross-checked by two researchers, and any discrepancies were resolved by a third researcher.

**Table 1:** Characteristics and quality assessment of included literature

		Bismuth-Corlette (I:II:III;IV)		Sample size		Age		
Study	Country	Open Surgery	Robotic Surgery	Open Surgery	Robotic Surgery	Open Surgery	Robotic Surgery	NOS score
Chou 2020^[11]^	China	4:13:14:0	1:10:5:0	31	16	60.0±9.0	68.0±7.0	7
Xu 2016^[12]^	China	0:4:17:11	0:1:5:4	32	10	59 (37-77)	54 (36–77)	6
Chen 2012^[13]^	China	4:10:15:11	7:13:19:12	40	51	52.6±11.4	62.1+11.7	9
Sebastian 2023^[14]^	America	NA	NA	62	25	65 (29–81)	61 (27–79)	7

### Evidence quality evaluation

The Grading of Recommendations Assessment, Development and Evaluation (GRADE) system is used to evaluate the quality of outcome indicators^10^. The following five factors in the GRADE system may affect the quality of research evidence: (1) risk of bias; (2) inconsistency; (3) accuracy; (4) indirectness; (5) publication bias. Divide the quality of outcome indicator evidence into high (not downgraded), medium (downgraded by 1 level), low (downgraded by 2 levels), and extremely low (downgraded by 3 levels).

### Statistical analysis

Statistical processing: statistical analysis was performed using RevMan 5.4 software. Continuous variables and binary variables were compared utilizing odds ratio (OR), mean difference (MD), and their respective 95% CI. Heterogeneity within the study was assessed using *I* ^2^: If *I* ^2^≤50%, indicating low heterogeneity, the fixed-effect model was utilized for analysis; If *I* ^2^>50%, indicating significant heterogeneity, the random-effects model was employed for analysis. A funnel plot was utilized to assess potential publication bias. Statistical significance was determined at *P*<0.05.

## Results

### Study selection and characteristics

**Figure 3:**
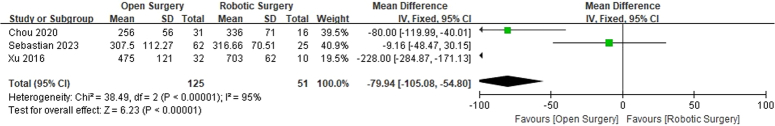
A comparison of operation time between robotic surgery and open surgery for hilar cholangiocarcinoma.

Following the established search strategy, we retrieved a total of 148 articles. Following screening based on the established criteria, four relevant articles met the criteria for inclusion. The literature screening process is depicted in Figure 1. All four articles were case–control studies, involving a total of 267 patients: 177 males and 90 females, with an average age of (58.8±5.7) years. Based on the mode of operation, 165 patients underwent open surgery, while 102 patients underwent robotic surgery. The basic characteristics and quality scores of the included literature are outlined in Table 2 and Figure 3.

**Table 2:** Characteristics and quality assessment of included literature.

		Bismuth–Corlette (I:II:III;IV)		Sample size		Age		
Study	Country	Open surgery	Robotic surgery	Open surgery	Robotic surgery	Open surgery	Robotic surgery	NOS score
Chou 2020^11^	China	4:13:14:0	1:10:5:0	31	16	60.0±9.0	68.0±7.0	7
Xu 2016^12^	China	0:4:17:11	0:1:5:4	32	10	59 (37–77)	54 (36–77)	6
Chen 2012^13^	China	4:10:15:11	7:13:19:12	40	51	52.6±11.4	62.1+11.7	9
Sebastian 2023^14^	America	NA	NA	62	25	65 (29–81)	61 (27–79)	7

### Operation time

**Figure 4:**
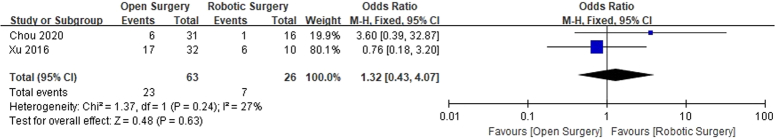
A Comparison of Intraoperative blood transfusion rate between robotic surgery and open surgery for Hilar Cholangiocarcinoma.

Operation time data was available in three references^11–13^, and significant heterogeneity (*I* ^2^=95%) was observed among the studies. The random-effects model was utilized for pooling. The results of the meta-analysis indicated that there was no significant difference found in operation time between the robot operation group and the open surgery group (MD=−103.96, 95% CI: −216.90–8.98, *P*=0.070; Fig. 4).

### Intraoperative blood transfusion rate

**Figure 5:**
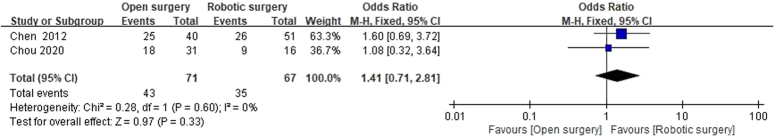
A comparison of R0 excision rate between robotic surgery and open surgery for hilar cholangiocarcinoma.

Intraoperative blood transfusion rate data was available in two references^9,10^, and no significant heterogeneity was observed among the studies (*I* ^2^=27%). The fixed-effects model was utilized for pooling. The results indicated that there was no significant difference found in the intraoperative blood transfusion rate between the robot operation group and the open surgery group (OR=1.32, 95% CI: 0.43–4.07, *P*=0.630; Fig. 5).

### R0 excision rate

**Figure 6:**
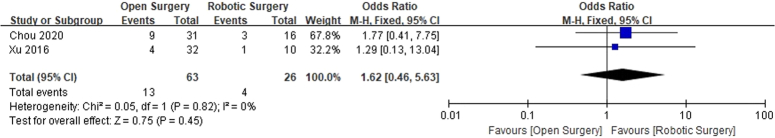
A comparison of Lymph Node Metastasis Rate between robotic surgery and open surgery for for hilar cholangiocarcinoma.

Two studies^11,13^ reported the R0 resection rate, and no significant heterogeneity was observed among the studies (*I* ^2^=0%). A fixed-effects model was utilized for pooling. The results indicated that there was no significant difference found in the R0 resection rate between the robot operation group and the open operation group (OR=1.41, 95% CI: 0.71–2.81, *P*=0.330; Fig. 6).

### Lymph node metastasis rate

**Figure 7:**
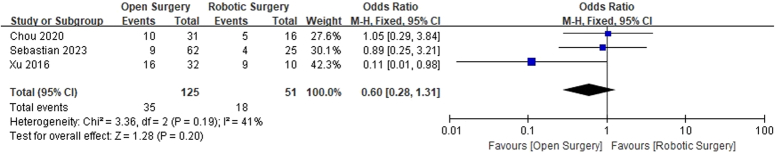
A comparison of Incidence of postoperative complications between robotic surgery and open surgery for hilar cholangiocarcinoma.

Two studies^11,12^ reported the lymph node metastasis rate, and no significant heterogeneity was observed among the studies (*I* ^2^=0%). The fixed-effects model was utilized for pooling. The results of the meta-analysis indicated that there was no significant difference found in the rate of lymph node metastasis between the robotic operation group and the open operation group (OR=1.62, 95% CI: 0.46–5.63, *P*=0.450; Fig. 7).

### Incidence of postoperative complications

**Figure 8:**
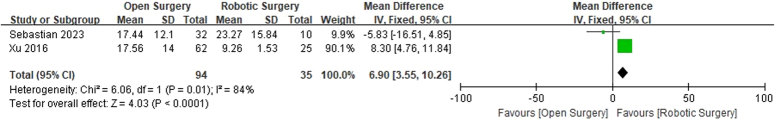
A comparison of Postoperative hospitalization time between robotic surgery and open surgery for hilar cholangiocarcinoma.

Postoperative complications were reported in three studies^11,12,14^, and no significant heterogeneity was observed among the studies (*I* ^2^=41%). The fixed-effects model was utilized for pooling. The results indicated that there was no significant difference found in the incidence of postoperative complications between the robotic operation group and the open operation group (OR=0.60, 95% CI: 0.28–1.31, *P*=0.200; Fig. 8). A funnel plot was employed to assess publication bias among the studies reporting postoperative complications. The funnel plot demonstrated that all the included studies fell within the 95% CI, suggesting no significant publication bias.

### Postoperative hospitalization time

**Figure 9:**
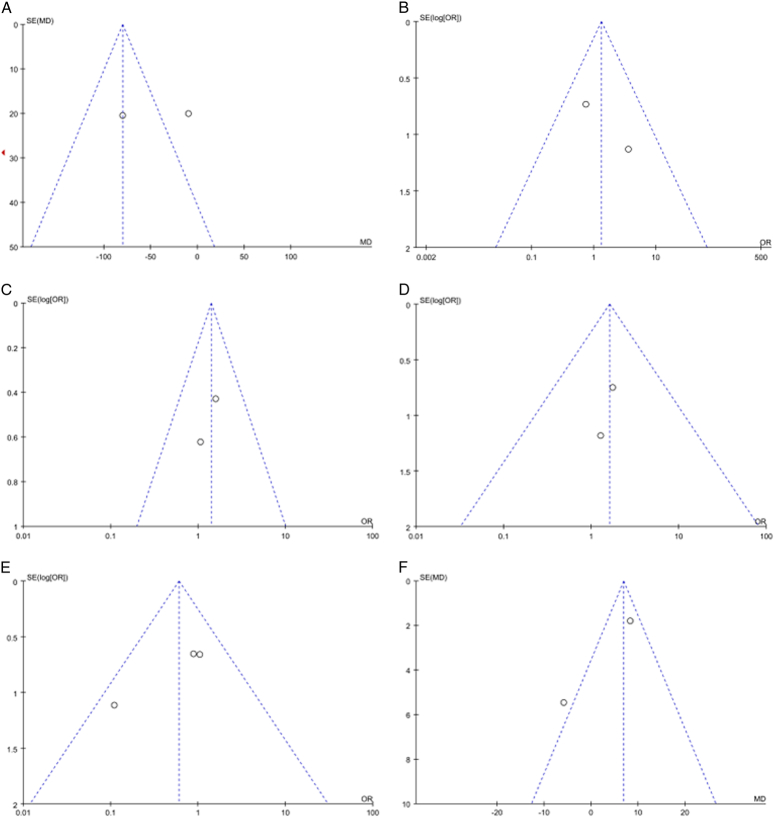
Funnel plots of meta-analysis of robotic and open surgery for hilar cholangiocarcinoma. Note: Figure A: funnel plot of operative time. Figure B: funnel plot of intraoperative blood transfusion rate. Figure C: funnel plot of R0 removal rate. Figure D: funnel plot of lymph node metastasis rate. Figure E: funnel plot of postoperative complications. Figure F: funnel plot of postoperative hospital stay.

Postoperative hospitalization time was reported in two studies^12–14^, and significant heterogeneity was observed among the studies (*I* ^2^=84%). The random-effects model was utilized for pooling. The results of the meta-analysis indicated that there was no significant difference found in postoperative hospital stay between the robot operation group and the open operation group (OR=2.17, 95% CI: −11.56–15.90, P=0.760; Fig. 9).

### Subgroup analysis based on Bismuth–Corllett typing

**Figure 10:**
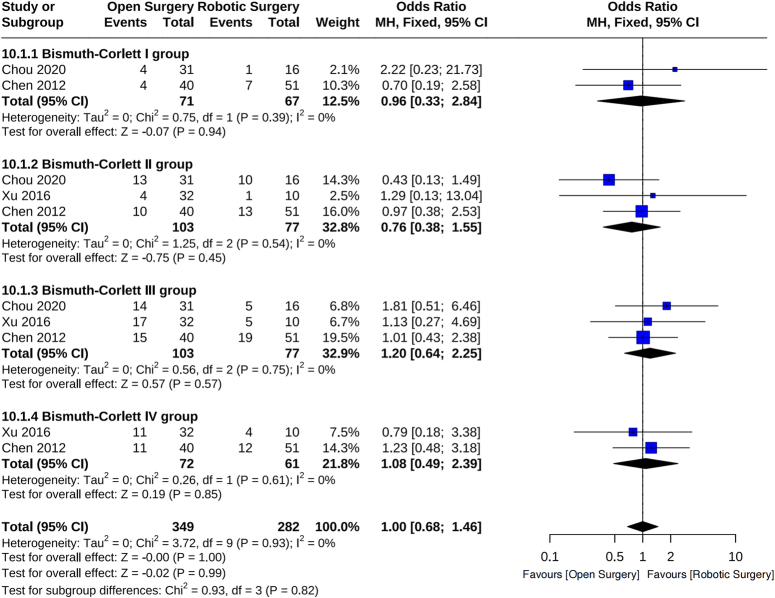
Subgroup analysis based on Bismuth–Corllett typing.

The Bismuth–Corlett classification, introduced by Bismuth in 1975^15^ and subsequently revised in 1992^16^, stands as the predominant method for categorizing hilar cholangiocarcinoma within clinical spheres. This classification, grounded in preoperative imaging, intraoperative tumor localization, and postoperative pathological delineations, delineates the condition into four distinct types. In our study’s subgroup analysis, it emerged that Bismuth–Corlett II and III constituted the most prevalent subtypes, representing 32.8 and 32.9% of cases, respectively (see Fig. 10). Remarkably, this finding parallels the conclusions drawn by Chaitererakij *et al*.^17^ from the Mayo Clinic in the United States. Their retrospective analysis, encompassing 413 patients with hilar cholangiocarcinoma, delineated the distribution across stages within the resection and palliative biliary drainage cohorts. Notably, in the resection group (*n*=82), patients were distributed as follows: 32 in stage I, 26 in stage II, and 16 in stage III. Conversely, among the 224 patients undergoing palliative biliary drainage, the distribution was as follows: 11 in stage I, 34 in stage II, 119 in stage III, and 54 in stage IV. Furthermore, their investigation revealed median survival times of 48.6, 21.8, 8.6, and 2.8 months for patients in stages I, II, III, and IV, respectively. These findings carry notable implications for tailoring surgical strategies to patients presenting with varying Bismuth subtypes.

### Publish bias analysis

To assess publication bias, a funnel plot was generated, utilizing the incidence of postoperative complications as an exemplar (refer to Fig. 9E). The resulting funnel plot depicted three scatter plots, each corresponding to one of the three articles ultimately included in the meta-analysis. Notably, all three scatter plots were positioned in the upper-middle region of the funnel plot and displayed a roughly symmetric distribution. This pattern suggests the presence of publication bias within the included studies, albeit with a minimal likelihood. Consequently, the potential impact of publication bias on the meta-analysis results is considered marginal.

## Discussion

The potential of robotic surgery in treating liver tumors lies in its minimally invasive nature and swift recovery. In the study by Giulianotti *et al*.^5^, the utilization of the Leonardo da Vinci robot facilitated procedures like right hepatectomy, Roux-en-Y choledochojejunostomy, and biliary reconstruction for patients with hilar cholangiocarcinoma. Importantly, no fatalities were recorded postoperation, though a complication rate of 21.4% (15/70) was observed. This investigation suggests comparable efficacy and safety between machine-assisted surgery and traditional open surgery for treating hilar cholangiocarcinoma.

It is evident that the intricacy of addressing hilar cholangiocarcinoma involves precise hilar dissection, comprehensive lymph node dissection, and intricate biliary tract reconstruction^18–20^. Xu *et al*. presented findings from 10 patients with hilar cholangiocarcinoma who underwent robotic radical resection, noting an operation duration of (703±62) min and blood loss of (1360±809) ml. Additionally, Chucky *et al*.^9^ conducted a comparison between robotic surgery (16 patients) and open surgery (31 patients) for treating hilar cholangiocarcinoma. They reported an operation time of (336±71) min for the robot group and (256±56) min for the open surgery group. Moreover, the blood transfusion rate was notably lower in the robot group at 6.3% (1 /16) compared to 19.4% (6 / 31) in the open surgery group. It is worth noting that although the blood transfusion rate was lower in robotic surgery, the operation time was comparatively longer.

In this study, the comparable operation times between robotic and open surgery in hilar cholangiocarcinoma patients might be attributed to surgeons being at the initial stages of the robotic surgical learning curve^21^. The primary cause of bleeding during hilar cholangiocarcinoma resection often stems from inadvertent damage to adjacent blood vessels during the dissection and separation of surrounding tissues. Robotic surgery, with its magnified three-dimensional visual field, facilitates more precise vascular ligation and suturing, thereby mitigating blood loss^22,23^.

The surgical management of Bismuth–Corlette type II–III hilar cholangiocarcinoma has notably increased recently, involving procedures such as hemihepatectomy plus caudate lobectomy or hepatectomy, lymph node dissection, extrahepatic cholangiectomy, and Roux-en-Y choledochojejunostomy^24,25^. In this meta-analysis, the lymph node metastasis rate observed in patients undergoing robotic surgery appeared lower than that in patients undergoing open surgery, but this disparity lacked statistical significance (*P*>0.05). Moreover, there was no significant distinction in the R0 resection rate between robotic and open surgery, possibly due to the prevailing use of robotic systems in left hemihepatectomy procedures where limited space constraints could influence outcomes.

Certainly, previous reports have highlighted that when compared to open surgery, robotic surgery for hilar cholangiocarcinoma exhibits a lower incidence of postoperative complications, facilitates quicker recovery, and potentially enhances the patients’ quality of life^26,27^. However, within this meta-analysis, while we meticulously compared the incidence of postoperative complications between robotic and open surgery, no significant difference emerged between the two groups. In a study by Xu *et al*.^12^, a review and analysis of data from 10 cases of hilar cholangiocarcinoma treated via robotic surgery indicated bile leakage as the most prevalent postoperative complication. Early identification of such complications, coupled with enhanced surgeon suture skills and timely intervention (inclusive of abdominal drainage), holds crucial significance for patient management and recovery.

This meta-analysis bears certain limitations. Firstly, as all the included studies were case–control in nature and exhibited heterogeneity, the reliability of the analytical outcomes might be affected. Secondly, the limited number of included studies and the absence of assessments related to primary survival duration and tumor recurrence efficacy could impact the credibility of the analytical findings. Therefore, future research endeavors should aim at conducting more extensive, multicenter clinical studies with larger sample sizes to substantiate and reinforce the conclusions drawn in this study.

## Ethical approval

The study is a meta-analysis and does not involve ethical approval, was reported in accordance with PRISMA (Preferred Reporting Items for Systematic Reviews and Meta-Analyses) guidelines.

## Consent

The study, which is a meta-analysis and does not require ethics committee approval and fully informed written consent, has been reported in accordance with PRISMA (Preferred Reporting Items for Systematic Reviews and Meta-Analyses) guidelines.

## Source of funding

This work was supported by the Yunnan Fundamental Research Projects (Grant No.202101AT070239). The corresponding author of this article, Huang Jie, is the first person in charge of this fund and participated in the whole process of data collection, analysis and interpretation.

## Author contribution

J.H.: designed this study; M.H. and J.H.: wrote the paper, participated in article quality control, and full text review; D.X. and Y.Z.: participated in literature quality, evaluation, and statistical mapping; A.L. and X.L.: involved in literature retrieval, literature screening, and data extraction. All authors contributed to the article and approved the submitted version.

## Conflicts of interest disclosure

The authors declare that the research was conducted in the absence of any commercial or financial relationships that could be construed as a potential conflict of interest.

## Research registration unique identifying number (UIN)

The meta-analysis has been registered on the Prospero platform with the following certification details: ID=CRD42024527511. The status of the registration with PROSPERO is ‘Review Completed not published’. https://www.crd.york.ac.uk/prospero/.

## Guarantor

Manqin Hu.

## Data availability statement

All data supporting the findings of this study are available within the paper and its Supplementary. The experimental data that support the findings of this study are openly available in PubMed, Embase, WOS, CNKI, and Wangfa data.

## Provenance and peer review

Not commissioned, externally peer-reviewed.

## References

[1] Mansour JC Aloia TA Crane CH. Hilar cholangiocarcinoma: expert consensus statement. HPB 2015;17:691–699. 26172136 10.1111/hpb.12450 PMC4527854

[2] Han ZL Peng JF Niu T. Advances in surgical treatment of hilar cholangiocarcinoma. Chin J Hepatobiliary Surg 2023;29:71–75.

[3] Matsuyama R Morioka D Mori R. Our rationale of initiating neoadjuvant chemotherapy for hilar cholangiocarcinoma: a proposal of criteria for “Borderline Resectable” in the field of surgery for hilar cholangiocarcinoma. World J Surg 2019;43:1094–1104. 30536024 10.1007/s00268-018-04883-y

[4] Ito F Cho CS Rikkers LF. Hilar cholangiocarcinoma: current management. Ann Surg 2009;250:210–218. 19638920 10.1097/SLA.0b013e3181afe0ab

[5] Giulianotti PC Coratti A Sbrana F. Robotic liver surgery: results for 70 resections. Surgery 2011;149:29–39. 20570305 10.1016/j.surg.2010.04.002

[6] Zhang T Zhao ZM Gao YX. The learning curve for a surgeon in robot-assisted laparoscopic pancreaticoduodenectomy: a retrospective study in a high-volume pancreatic center. Surg Endosc 2019;33:2927–2933. 30483970 10.1007/s00464-018-6595-0

[7] Page MJ McKenzie JE Bossuyt PM. The PRISMA 2020 statement: an updated guideline for reporting systematic reviews. Int J Surg 2021;88:105906. 33789826 10.1016/j.ijsu.2021.105906

[8] Shea BJ Reeves BC Wells G. AMSTAR 2: a critical appraisal tool for systematic reviews that include randomised or non-randomised studies of healthcare interventions, or both. BMJ (Clinical research ed) 2017;358:j4008. 10.1136/bmj.j4008 PMC5833365 28935701

[9] Stang A. Critical evaluation of the Newcastle-Ottawa scale for the assessment of the quality of nonrandomized studies in meta-analyses. Eur J Epidemiol 2010;25:603–605. 20652370 10.1007/s10654-010-9491-z

[10] Mendoza C Kraemer P Herrera P. [Clinical guidelines using the GRADE system (Grading of Recommendations Assessment, Development and Evaluation)]. Rev Med Chil 2017;145:1463–1470. 29664529 10.4067/s0034-98872017001101463

[11] Chou S Chang ZY Zhao GD. Robotic hilar cholangiocarcinoma radical resection compared with laparotomy in prognosis. Chinese J Surg 2020;58:230–234. 10.3760/cma.j.issn.0529-5815.2020.03.012 32187928

[12] Xu Y Wang H Ji W. Robotic radical resection for hilar cholangiocarcinoma: perioperative and long-term outcomes of an initial series. Surg Endoscopy 2016;30:3060–3070. 10.1007/s00464-016-4925-7 27194255

[13] Chen ZF. Comparative study on the treatment of Hilar cholangilarcinoma by Da Vinci Robotic Surgical System and Objective Traditional Open Surgery [D]. Shanxi Medical University; 2012.

[14] Knitter S Feldbrügge L Nevermann N. Robotic versus laparoscopic versus open major hepatectomy–an analysis of costs and postoperative outcomes in a single-center setting. Langenbeck’s Arch Surg 2023;408:214. 37247050 10.1007/s00423-023-02953-x PMC10226911

[15] Bismuth H Corlette MB. Intrahepatic cholangioenteric anastomosis in carcinoma of the hilus of the liver. Surg Gynecol Obstetr 1975;140:170–178. 1079096

[16] Bismuth H Nakache R Diamond T. Management strategies in resection for hilar cholangiocarcinoma. Ann Surg 1992;215:31–38. 1309988 10.1097/00000658-199201000-00005 PMC1242367

[17] Chaiteerakij R Harmsen WS Marrero CR. A new clinically based staging system for perihilar cholangiocarcinoma. Am J Gastroenterol 2014;109:1881–1890. 25384902 10.1038/ajg.2014.327 PMC4341961

[18] Valle JW Borbath I Khan SA. Biliary cancer: ESMO Clinical Practice Guidelines for diagnosis, treatment and follow-up. Ann Oncol 2016;27:v28–v37. 27664259 10.1093/annonc/mdw324

[19] Li LZ Yang ZS Li K. Efficacy of robot-assisted laparoscopic resection for choledochal cysts. Chin J Hepatobiliary Surg 2022;28:898–901.

[20] Wang W Fei Y Liu J. Laparoscopic surgery and robotic surgery for hilar cholangiocarcinoma: an updated systematic review. ANZ J Surg 2021;91:42–48. 32395906 10.1111/ans.15948

[21] Kamarajah SK Bundred J Manas D. Robotic versus conventional laparoscopic liver resections: a systematic review and meta-analysis. Scandinav J Surg 2021;110:290–300. 32762406 10.1177/1457496920925637

[22] Giulianotti PC Quadri P Durgam S. Reconstruction/repair of iatrogenic biliary injuries: is the robot offering a new option? Short clinical report. Ann Surg 2018;267:e7–e9. 28657946 10.1097/SLA.0000000000002343

[23] Lim C Salloum C Tudisco A. Short-and long-term outcomes after robotic and laparoscopic liver resection for malignancies: a propensity score-matched study. World J Surg 2019;43:1594–1603. 30706105 10.1007/s00268-019-04927-x

[24] Cillo U D’Amico FE Furlanetto A. Robotic hepatectomy and biliary reconstruction for perihilar cholangiocarcinoma: a pioneer western case series. Updates Surg 2021;73:999–1006. 33861401 10.1007/s13304-021-01041-3 PMC8184707

[25] Tee MC Brahmbhatt RD Franko J. Robotic resection of type I hilar cholangiocarcinoma with intrapancreatic bile duct dissection. Ann Surg Oncol 2022;29:964–969. 34613533 10.1245/s10434-021-10811-7

[26] Yu YB Jin Q Song Y. Short-term effect of robotic pancreaticoduodenectomy and laparoscopic pancreaticoduodenectomy: a meta-analysis. Chin J Hepatobiliary Surg 2021;27:211–214.

[27] Machado MA Mattos BV Lobo Filho MM. Robotic resection of hilar cholangiocarcinoma. Ann Surg Oncol 2020;27:4166–4170. 32363511 10.1245/s10434-020-08514-6

